# The rise of tuberculosis: regression in combating advances as a legacy of COVID-19?

**DOI:** 10.31744/einstein_journal/2022CE0196

**Published:** 2022-11-09

**Authors:** Gabriel Martins de Barros

**Affiliations:** 1 Universidade Federal do Piauí Teresina PI Brazil Universidade Federal do Piauí, Teresina, PI, Brazil.

Dear Editor,

Research indicates that in the beginning of the COVID-19 pandemic there was a decrease in number of tuberculosis diagnosis. This reduction has been suggested to have occurred due to social distancing and more constant hygiene routines, such as hand washing and sanitizing, as well as continued use of masks.^(1,2)^

However, so far it is unclear whether social distancing may have contributed to reduce tuberculosis or HIV transmission. Perhaps, for the former, the longer time spent indoors in crowded households may have increased domestic transmission of tuberculosis.^(3)^

In addition, given that the focus was on COVID-19, resources, research, and drugs to tuberculosis were constrained.^(1–3)^ In Brazil, a country with a high endemic load, data revealed an increase in the number of treatment dropouts and in the number of deaths from tuberculosis.^(2–4)^ Other countries also reported an increase in the number of consultations and a decrease in confirmed tuberculosis cases during the pandemic. These facts may indicate that there was a failure in screening for this disease.^(2)^

The correlation between the diseases may have mechanisms involved to be studied, an experimental model showed that MHV-1 coronavirus (murine coronavirus) activates an altruistic defense mechanism mediated by stem cells that reactivates latent tuberculosis: the lung infection exhibited viral loads 20 times lower than control mice without *Mycobacterium tuber culosis*.^(5)^

It is worth mentioning that tuberculosis can remain latent for decades, therefore, this disease has a risk of persistent reactivation. Latent tuberculosis reactivation results from a failure of immune surveillance, mainly due to immunosuppressive therapy or HIV infection.^(6)^ In a recent systematic review on the prevalence of latent tuberculosis, the global burden derived from tuberculin skin testing and interferon y-releasing trials. The prevalence of tuberculosis reported was 24.8%, which may indicate that a quarter of the population is infected.^(7)^

The literature also shows that HIV screening was also affected by the pandemic,^(3)^ as well as various steroids and monoclonal antibodies used to control symptoms of COVID-19. Health services still need to consider the stigma and fear of individuals of being misdiagnosed with COVID-19 due to the similarity of symptoms, which impact the demand for care.^(8)^

Finally, poor eating habits and inadequate nutrition associated with alcohol abuse, bad habits that become common during the pandemic, can lead to immune compromising and increased risks to tuberculosis,^(3,6)^ especially in low- and middle-income countries.

As a legacy, the COVID-19 pandemic will have direct and indirect effects on tuberculosis, such as the reduction or interruption of treatment, screening, notification and investment in inputs and programs, particularly in low- and middle-income countries. To prevent the resurgence of an old pathogen and to reduce the burden of preventable deaths, governments and researchers need to take a special look at tuberculosis in order to guarantee the appropriated measures for the prevention, diagnosis and treatment of this disease. These efforts are special important to respond to the future phases of COVID-19.
